# Structural changes and expression of hepatic fibrosis-related proteins in coculture of *Echinococcus multilocularis* protoscoleces and human hepatic stellate cells

**DOI:** 10.1186/s13071-021-05037-1

**Published:** 2021-12-02

**Authors:** Deping Cao, Emad Shamsan, Bofan Jiang, Haining Fan, Yaogang Zhang, Mustafa Abdo Saif Dehwah

**Affiliations:** 1 Department of Human Parasitology, Guilin Medical University, Guilin, 541199 Guangxi Zhuang China; 2 Department of Immunology, Faculty of Medicine, Qinghai University, Xining, 810001 Qinghai China; 3 The Key Echinococcosis Laboratory, Qinghai University Affiliated Hospital, Xining, 810001 Qinghai China; 4 Department of Hepatobiliary and Pancreatic Surgery, Qinghai University Affiliated Hospital, Xining, 810001 Qinghai China; 5 Laboratories Department, Faculty of Medical Sciences, Taiz University, Turba Branch, 70270 Taiz, Yemen

**Keywords:** *Echinococcus multilocularis*, Hepatic stellate cell, Hepatic fibrosis, Protoscoleces, Collagen-I, Alpha-smooth muscle actin, Osteopontin

## Abstract

**Background:**

*Echinococcus multilocularis* is the causative agent of human hepatic alveolar echinococcosis (AE). AE can cause damage to several organs, primarily the liver, and have severe outcomes, such as hepatic failure and encephalopathy. The main purpose of this study was to explore the interactions between hepatic stellate cells (HSCs) and *E. multilocularis* protoscoleces (PSCs). The results of this study provide an experimental basis for further examination of the pathogenesis of hepatic fibrosis due to AE infection.

**Methods:**

We investigated the role of *Echinococcus multilocularis* (Echinococcus genus) PSCs in hepatic fibrosis by examining structural changes and measuring hepatic fibrosis-related protein levels in cocultures of PSCs and human HSCs. Structural changes were detected by transmission electron microscopy (TEM), and levels of the hepatic fibrosis-related proteins collagen I (Col-I), alpha-smooth muscle actin (α-SMA) and osteopontin (OPN) were measured by western blotting and enzyme-linked immunosorbent assay (ELISA).

**Results:**

Under coculture (1) both PSCs and HSCs exhibited morphological changes, as observed by TEM; (2) Col-I, α-SMA, and OPN expression levels, which were determined by western blotting and ELISA, significantly increased after 3 days of incubation.

**Conclusions:**

The results of this study provide insights into the molecular mechanisms of AE-induced hepatic fibrosis.

**Graphical abstract:**

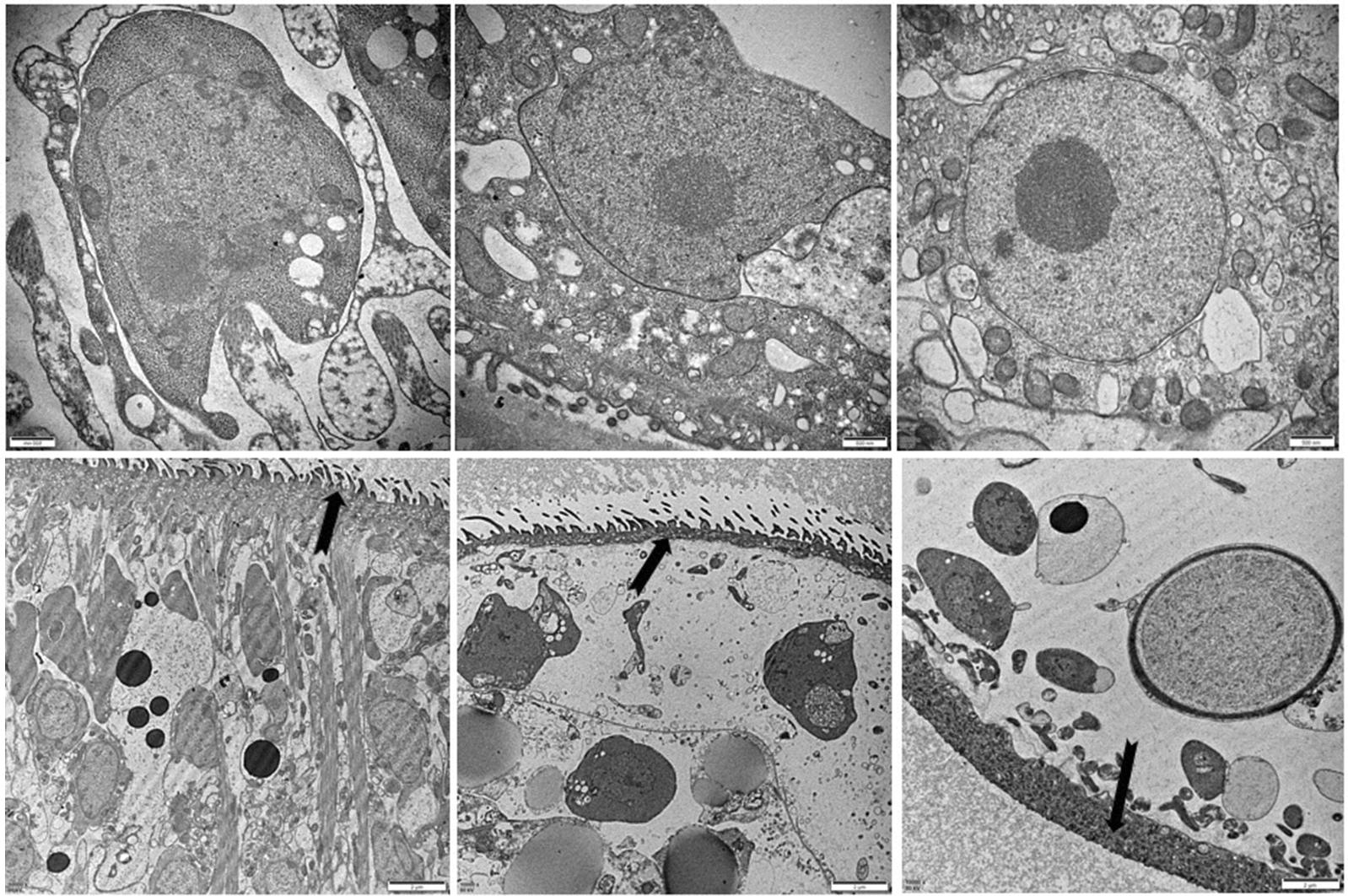

## Background

Alveolar echinococcosis (AE), one of the deadliest human diseases, is caused by *Echinococcus multilocularis* [1, 2] and is prevalent in most of the northern hemisphere [3, 4]. Epidemiological surveys have shown that AE is common in central Asia, including areas of Kyrgyzstan, Kazakhstan, and northwest China [5, 6]. Protoscoleces (PSCs) of *E. multilocularis* invade the liver and trigger hepatic fibrosis. Hepatic cells at this phase of the disease are likely damaged by toxic products of the metacestodes [7]. Peri-parasite granulomas form in the liver and cause irreversible hepatic fibrosis, which makes surgical resection difficult, and secondary infections typically occur [8]. Labsi et al. [9, 10] found that aqueous extract of* Punica granatum* peel and IL-17A are good candidate treatments for cystic hydatidosis in humans. Patients with AE can remain asymptomatic for up to 15 years [11], and AE is often at a late stage when patients are first seen at clinic. AE is fatal in 94% of cases, particular when it has not been diagnosed and the patient treated [12, 13]. The oncosphere produced during AE grows like a tumour, which is why it is also referred to as “worm cancer”. It is impossible to completely remove the tumour because of its growth. Therefore, understanding the mechanisms underlying the interactions between the parasite and humans and the pathogenesis of AE is necessary to develop treatments for echinococcosis-induced liver damage.

Hepatic stellate cells (HSCs), which are the major cells responsible for the formation of extracellular matrix (ECM) proteins during cirrhosis, are found in the space of Disse and act as a major storage site for vitamin A [14, 15]. They are stimulated in response to certain growth factors, inflammatory stimuli, and, in the case of liver damage, oxidative stress. For example, damaged liver cells, resident phagocytic cells, infiltrating inflammatory cells, aggregated platelets, and Kupffer cells can activate HSCs. Activated HSCs differentiate into muscle fibroblasts, which express alpha-smooth muscle actin (α-SMA) and accelerate the process of cirrhosis. Under the pathological conditions of cirrhosis, HSCs lose their retinoid (vitamin A) and synthesize a large quantity of ECM components, including collagen, proteoglycans and glycosaminoglycans [16–18]. They also proliferate and migrate, and in addition to the ECM components they generate, such as fibronectin, fibrillar collagen and the aforementioned proteoglycans, lead to the formation of septa in the chronically damaged liver [19]. Moreover, an imbalance in the formation of collagen fibres can cause fibrosis [20]. Hepatic parasitic fibrosis caused by *E. multilocularis* PSCs is a host response associated with immune cell infiltration which activates the differentiation of HSCs into fibroblasts [21]. Therefore, identifying the mechanisms underlying AE-induced liver fibrosis may help us to understand the pathogenesis of AE and develop better treatments for this disease.

In this study, *E. multilocularis* PSCs were cultured in modified media to investigate their role in liver fibrosis. The HSC–LX2 cell line was cocultured with PSCs, after which liver fibrosis-related proteins collagen I (Col-I), α-SMA, and osteopontin (OPN) were detected and their levels measured.

## Methods

### Isolation and cultured the *E. multilocularis* protoscoleces

Mongolian gerbils (males, 1 month old, 30 g weight) were infected with *E. multilocularis* PSCs and then killed with CO_2_ after 6 months. The livers were collected from the dead gerbils and washed with aseptic phosphate-buffered saline (PBS) under sterile conditions. After separating out the AE cysts, they were mashed into small pieces and passed through an aseptic sieve (mesh size 300 µm) to collect the PSCs. The PSCs were washed five times with PBS containing 1000 μg/mL streptomycin and 1000 U/mL penicillin. The PSCs were then placed in solution at room temperature (RT; 25–30 ℃) for 5 min and debris removed. The viability of the PSCs was tested by staining with 0.1% trypan blue, as dead PSCs were stained blue. Only PSCs showing > 90% viability were selected for further use [22]. PSCs were cultured in Dulbecco’s modified Eagle’s medium (DMEM) containing 10% (volume/volume) fetal bovine serum (Gibco, Grand Island, NY), 0.45% (weight/volume) yeast extract, 0.4% (weight/volume) glucose, 1000 μg/mL streptomycin, and 1000 U/mL penicillin at 37 °C in the presence of 5% CO_2_.

### Coculture HSC-LX2 and PSC

HSC-LX2 were obtained from the Beijing University of Biological Sciences (Beijing, China). HSCs were preserved in DMEM supplemented with 10% fetal bovine serum containing 1000 U/mL penicillin G and 1000 µg/mL streptomycin. HSCs were plated on 60-mm plates at 1.5 × 10^5^ cells/plate and the plates were then divided into five groups. Three of these groups were exposed to PSCs and the other two were used as the controls (HSCs only and PSCs only). The three groups of HSCs were exposed to PSCs and were cultured at one of the following PSC:HSC ratios: 1:200, 2:200 and 3:200. All of the groups were incubated at 37 °C in the presence of 5% CO_2_ for 24, 48, and 72 h. The harvested HSC-LX2 were divided into two groups. Cells of the first group were centrifuged and then collected to measure the expression of Col-I, α-SMA, and OPN, and the coculture supernatant was collected to measure Col-I, α-SMA and OPN levels by enzyme-linked immunosorbent assay (ELISA). Cells of the second group were collected to analyse structural changes.

### Transmission electron microscopy

Cultured HSC-LX2 and PSCs were examined by transmission electron microscopy (TEM) to observe changes in cell morphology. PSC specimens for TEM were immersed in fixative (2.5% glutaraldehyde) after washing them three times with PBS. The HSC specimens were washed with PBS and trypsin was added to the cultured cells. The cells were collected and immersed in fixative (2.5% glutaraldehyde). Thereafter, the specimens were imaged by TEM (HT7700; Hitachi, Japan).

### Western blotting

Proteins were extracted from cultured cell lysates with phosphatase and protease inhibitor cocktails. Protein (20 µg) was separated from each sample by 6% or 10% sodium dodecyl sulphate polyacrylamide gel electrophoresis and transferred to a polyvinylidene difluoride membrane. The membranes were blocked using 5% non-fatty milk in PBS containing 0.1% Tween-20 for 1 h at RT, and were then incubated with primary antibodies overnight at 4 °C. Subsequently, the membranes were washed with PBS containing 0.1% Tween-20 three times and then incubated with secondary antibodies conjugated with horseradish peroxidase for 90 min at RT. Protein bands were detected using a Fluor-S MultiImager (Bio-Rad, Hercules, CA). The band density was measured using NHI Image software (version 1.53; NIH, Bethesda, MD); β-actin served as a loading control. The antibodies used in the analysis were anti-Col-I (ab90395), anti-α-SMA (ab7817) and anti-OPN (ab8448) (Abcam, Cambridge, UK).

### Measurement of Col-I, α-SMA, OPN in the supernatant

Col-I, α-SMA and OPN levels were measured using ELISA according to the manufacturer’s instructions (Cloud-Clone, USA). HSC-LX2 were exposed to different ratios of PSCs for 24, 48, 72, and 96 h. Next, the supernatant was collected and centrifuged at 1000×*g* for 20 min and the pellet collected. The supernatant (100 µL) was transferred to a new tube, and the levels of Col-I, α-SMA and OPN measured. The samples were then prepared and mixed with the standard sample in 96-well plates. The concentrations of Col-I, α-SMA and OPN were determined by measuring the optical density at 450 nm using a spectrophotometer. Total levels of Col-I, α-SMA and OPN are expressed as nanograms per millilitre of protein.

### Statistical analysis

Data are presented as the mean ± SD. Data were assessed using GraphPad Prism 8.0 software (GraphPad, Inc., La Jolla, CA, USA) and one-way analysis of variance followed by Dunnett's various comparisons test.

## Results

### HSC-LX2 promoted the growth of PSCs

**Fig. 1:**
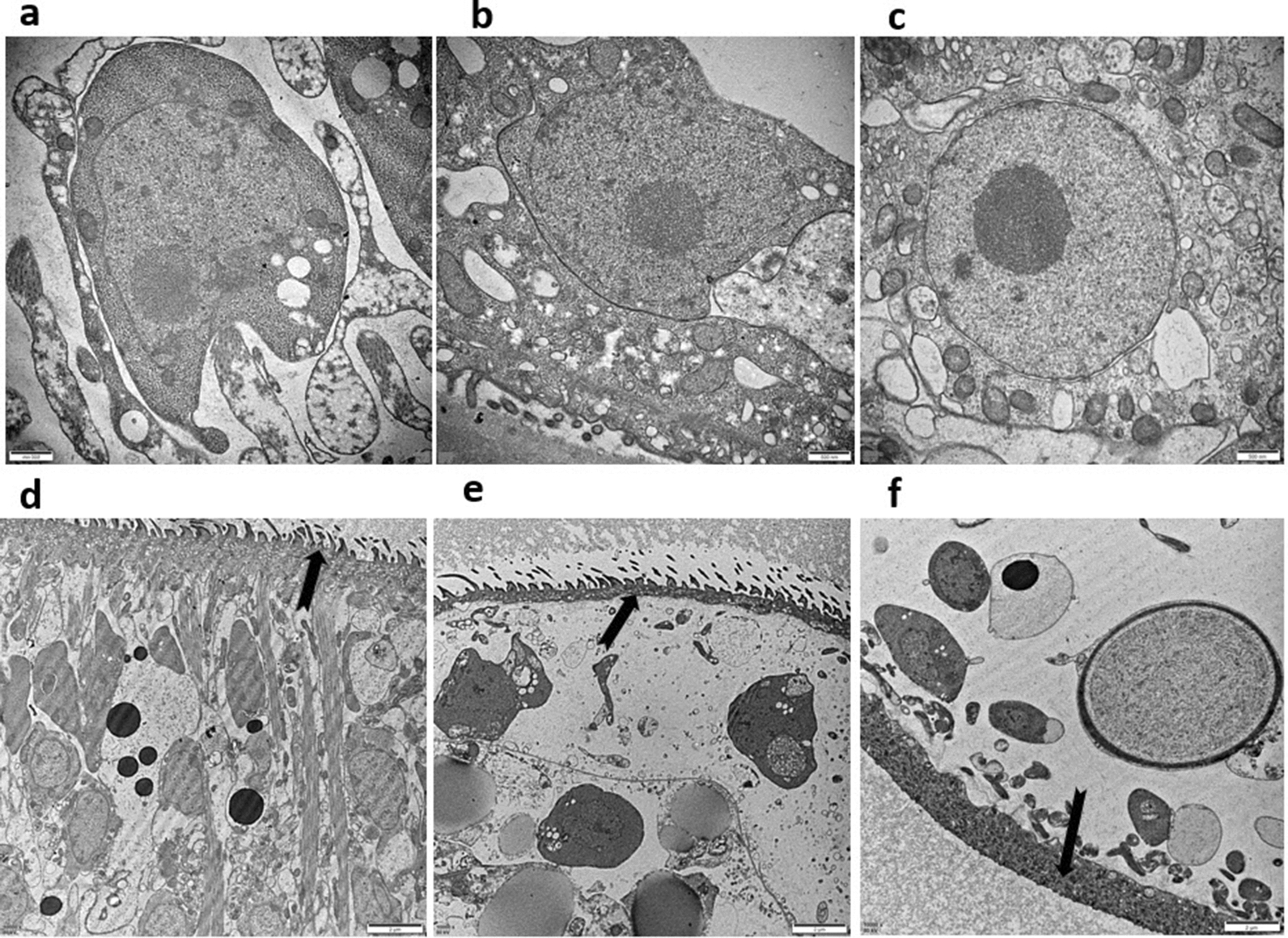
**a**–**f** Transmission electron microscopy (TEM, **a**–**c**: 30 000×; **d**–**f**: 10 000×) images of the internal structure of *Echinococcus multilocularis* protoscoleces (PSCs). **a** Normal cell structure of *E. multilocularis* PSC on co-culture 0 day. **b** Cell structure of *E. multilocularis* PSC on co-culture the day 4. vacuoles appearing in the cytoplasm. **c** Cell structure of *E. multilocularis* PSC on co-culture the day 4. the perinuclear space widening and mitochondria swelling and vacuoles appearing in the cytoplasm. **d** the microcilia on wall of *E. multilocularis* PSC were clearly visible on the 0 day (arrow). The microcilia on wall of *E. multilocularis* PSC become shorter and cell structure disappearing on the day 3 (**e**; arrow). The microcilia on wall of *E. multilocularis* PSC are disappeared and cell structure dissolved on the day 4 (**f**; arrow)

PSCs and HSC-LX2 were cocultured in DMEM for 3 days to examine their interaction. The PSCs became highly motile, developed rapidly and evaginated in the presence of HSC-LX2. TEM was used to observe the structural changes on day 3 in the PSCs cocultured with HSC-LX2. The micrographs showed a slight change in the walls of the PSCs cocultured with HSC-LX2, as the walls of the PSCs cocultured with HSC-LX2, as the microcilia on the walls of *E. multilocularis* PSCs become shorter and cell structure disappearing and dissolved on day 3 compared with those of the PSC control (Fig. 1a, d, e, f). Vacuoles appeared and the perinuclear space widening and mitochondria swelling in the cellss of PSCs occurred during co-culture on day 4 (Fig. 1b, c).

### Structural changes of HSC-LX2 in coculture with PSCs

**Fig. 2:**
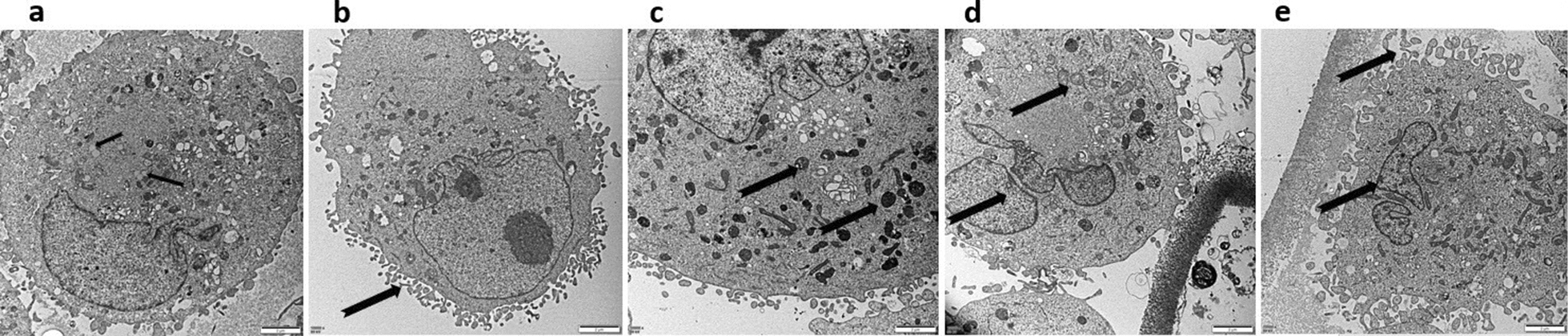
Structural characteristics of HSCs (TEM, 10 000 ×) (HSCs exposed to PSCs). **a** Lipid droplets are commonly seen in the cytoplasm in HSC on the 0 day (arrow). **b** Microvilli on the HSC clearly visible on the day 3(arrow). **c** Lipid droplets decreasing in cytoplasm; mitochondrial election density increasing and lysosomes increasing in cytoplasm in HSC (arrow). **d** The nuclei are malformed and the mitochondria are swelling on the day 3 (arrow). **e** The nuclei are irregular and microvilli on the cell membrane are increasing on the 4th day

**Fig. 3:**
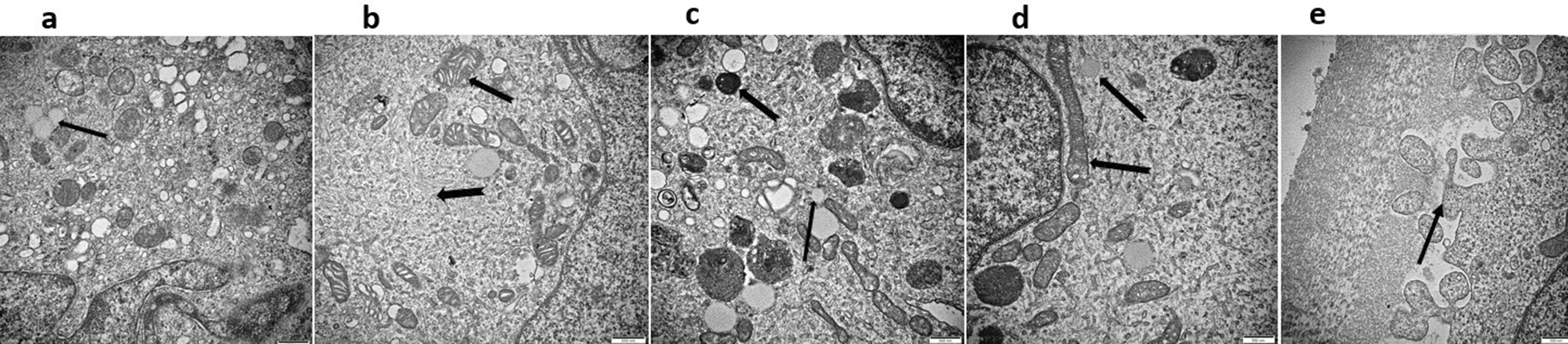
Structural characteristics of HSC (TEM, 30 000×) (HSCs exposed to PSCs). **a** The oval lipid droplets clearly visible in HSCs on 0 day. (arrow). **b**, **d** The mitochondrial cristae space widening and the microfilaments increasing in cytoplasm on the day 3 (arrow). **c** The lysosomes increasing and mitochondria swelling occurred in HSCs (arrow) on the day 3. **e** microvilli on the HSC cell membrane (arrow)

HSC-LX2 were activated at PSC:HSC-LX2 ratios of 1:200 and 2:200 for 3 days, but were inhibited at a PSC:HSC-LX2 ratio of 3:200. On day 3 of the coculture, structural changes were analysed by TEM. The lipid droplets that were detected by TEM in control HSC-LX2 on day 0 were present until day 3 (Fig. 2a; Fig. 3a). Lipid droplets were not observed in all the cells, but where they were observed in HSC-LX2 exposed to PSCs they had degenerated by day 3. This indicated that fibrogenesis had occurred (Fig. 2d; Fig. 3b). Furthermore, the lysosomes increasing and mitochondria swelling occurred in HSCs on the day 3 than normal HSC (Fig. 3c). Some changes in the cytoplasm were observed, such as the mitochondrial cristae space widening and the microfilaments increasing in HSC cytoplasm (Fig. 3d, e). The nuclei of HSCs cocultured wth PSCs also exhibited structural changes in comparison with those of normal control HSCs, as they are malformed or irregular, and microvilli on the cell membrane are increasing and the mitochondria are swelling on day 3 and 4 (Fig. 2d, e).

### Col-I expression in HSC-LX2 exposed to PSCs

**Fig. 4:**
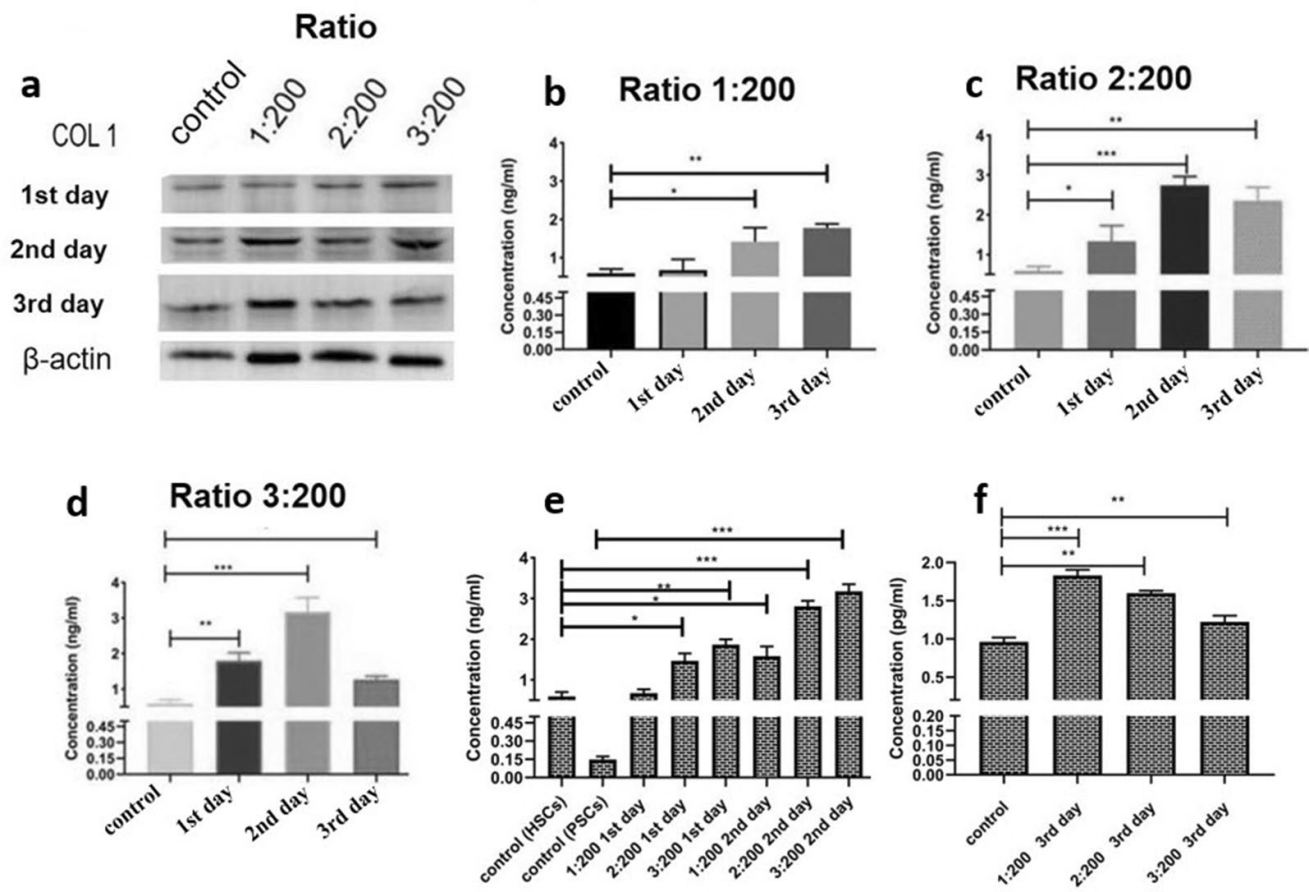
**a**–**f** Collagen I (*Col-I*) expression profile measured by western blotting (WB) and enzyme-linked immunosorbent assay (ELISA). **a** Expression of Col-I assessed by WB. **b** At a PSC:HSC-LX2 ratio of 1:200, Col-I expression had significantly increased by days 2 and 3 but not by day 1. **c** Col-I expression at a PSC:HSC-LX2 ratio of 2:200 was significantly higher on days 1, 2 and 3 compared to the control. **d** At a PSC:HSC-LX2 ratio of 3:200, Col-I expression had increased by days 1 and 2, but decreased by day 3. **e** Col-I expression increased in HSCs cocultured with PSCs for 48 h. **f** By day 3, Col-I expression had increased at a PSC:HSC-LX2 ratio of 1:200 but had started to decrease at PSC:HSC-LX2 ratios of 2:200 and 3:200. For other abbreviations, see Fig. 1

Col-I plays a significant role in liver fibrosis. Therefore, the expression of Col-I was examined in HSC-LX2 exposed to PSCs. The expression of Col-I at different ratios of PSC:HSC-LX2 (1:200, 2:200, and 3:200) was measured on days 1, 2 and 3 by western blotting (WB) (Fig. 4a). The Col-I level in the coculture supernatant was measured by ELISA. At a PSC:HSC-LX2 ratio of 1:200, the expression of Col-I had risen dramatically by days 2 (Dunnett’s various comparisons test, the following data processing by this statistical method, *q* = 3.83, *p* < 0.05) and 3 (*q* = 5.39, *p* < 0.01) (Fig. 4b, ANOVA: F_(3, 8)_ = 14.30, *p* = 0.0014). At a PSC:HSC-LX2 ratio of 2:200, the level of Col-I had significantly increased by days 1 (*q* = 3.37, *p* < 0.05), 2 (*q* =10.04, *p* < 0.001) and 3 (*q* = 7.18, *p* < 0.01) for the 2:200 ratio (Fig. 4c, ANOVA: F_(3, 8)_ = 38.49, *p* < 0.0001). At a PSC:HSC-LX2 ratio of 3:200, the elevation of Col-I was higher on day 2 (*q* = 5.15, *p* < 0.001) than on days 1 (*q* = 7.08, *p* < 0.01) and 3 (*q* = 4.66, *p* < 0.05) (Fig. 4d, ANOVA: F_(3, 8)_ = 42.01, *p* < 0.0001). Col-I expression levels had increased gradually by days 1 and 2 days at PSC:HSC-LX2 ratios of 1:200 and 2:200 (Fig. 4e, ANOVA: F_(10, 22)_ = 34.85, *p* < 0.0001). However, by day 3, at a ratio of 1:200, Col-I had continued to rise, but at ratios of 2:200 and 3:200 showed a decline (Fig. 4f, ANOVA: F_(3, 8)_ = 35.15, *p* < 0.0001).

### α-SMA expression in HSC-LX2 exposed to PSCs

**Fig. 5:**
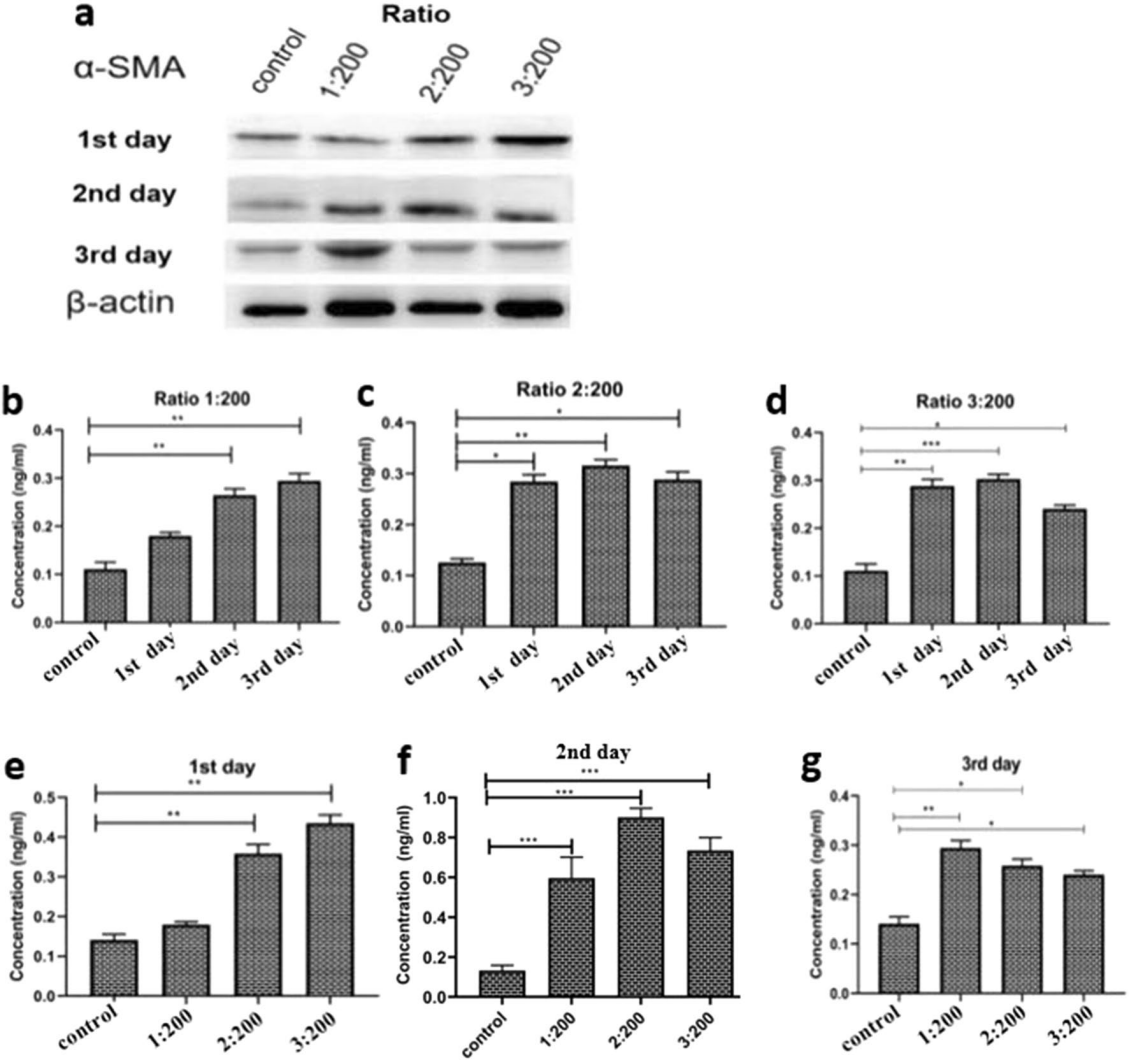
**a**–**g** Alpha-smooth muscle actin (α-*SMA*) expression profile measured by WB and ELISA. **a** α-SMA expression assessed by WB. **b** α-SMA expression was significantly higher on days 2 and 3 days, but was not on day 1 for the PSC:HSC-LX2 ratio of 1:200. **c** α-SMA expression was significantly higher on days 1, 2 and 3 for the PSC:HSC-LX2 ratio of 2:200. **d** For the PSC:HSC-LX2 ratio of 3:200, α-SMA expression had increased by days 1 and 2 but decreased by day 3. **e** The expression of α-SMA in HSCs cocultured with PSCs for 24 h increased. **f** By day 2, α-SMA expression had increased at all ratios of PSC:HSC-LX2 (1:200, 2:200 and 3:200). **g** By day 3, α-SMA expression had increased at a PSC:HSC-LX2 ratio of 1:200 but had started to decrease at ratios of 2:200 and 3:200. For other abbreviations, see Fig. 1

PSCs have been reported to play an essential role in the activation and proliferation of HSCs in coculture accompanied by the expression of α-SMA. α-SMA expression in HSCs exposed to PSCs was measured by WB (Fig. 5a) and confirmed by ELISA (Fig. 5b–g). α-SMA expression increased significantly at a PSC:HSC-LX2 ratio of 1:200 on the day 2 (*q* = 7.54, *p* < 0.01) and 3 day (*q* = 9.69, *p* < 0.01) (Fig. 5b, ANOVA: F_(3, 8)_ = 47.12, *p* < 0.0001). A significant increase in α-SMA expression was observed for the 2:200 ratio on the days 1, 2 and 3 (*q* = 6.35, *p* < 0.05; *q* = 22.9, *p* < 0.01; and *q* = 13.12, *p* < 0.05, respectively) (Fig. 5c, ANOVA: F_(3, 8)_ = 188.0, *p* < 0.0001). Additionally, a significant elevation in α-SMA expression was observed for 3:200 on days 1, 2 and 3 (*q* = 8.29, *p* < 0.01; *q* = 16.67, *p* < 0.001 and *q* = 12.17, *p* < 0.05, respectively) (Fig. 5d, ANOVA: F_(3, 8)_ = 119.0, *p* < 0.0001). α-SMA expression on day 1 was significantly higher at the PSC:HSC-LX2 ratios of 2:200 and 3:200 but not at 1:200 (Fig. 5e, ANOVA: F_(3, 8)_ = 93.85, *p* < 0.0001). The expression of α–SMA on day 2 was significantly higher at a ratio of 2:200 followed by 1:200 and then 3:200 (Fig. 5f, ANOVA: F_(3, 8)_ = 73.37, *p* < 0.0001). On day 3, α-SMA expression was moderate at a PSC:HSC-LX2 ratio of 1:200 and had decreased at ratios of 2:200 and 3:200 (Fig. 5g, ANOVA: F_(3, 8)_ = 48.42, *p* < 0.0001).

### OPN Expression in HSC-LX2 exposed to PSCs

**Fig. 6:**
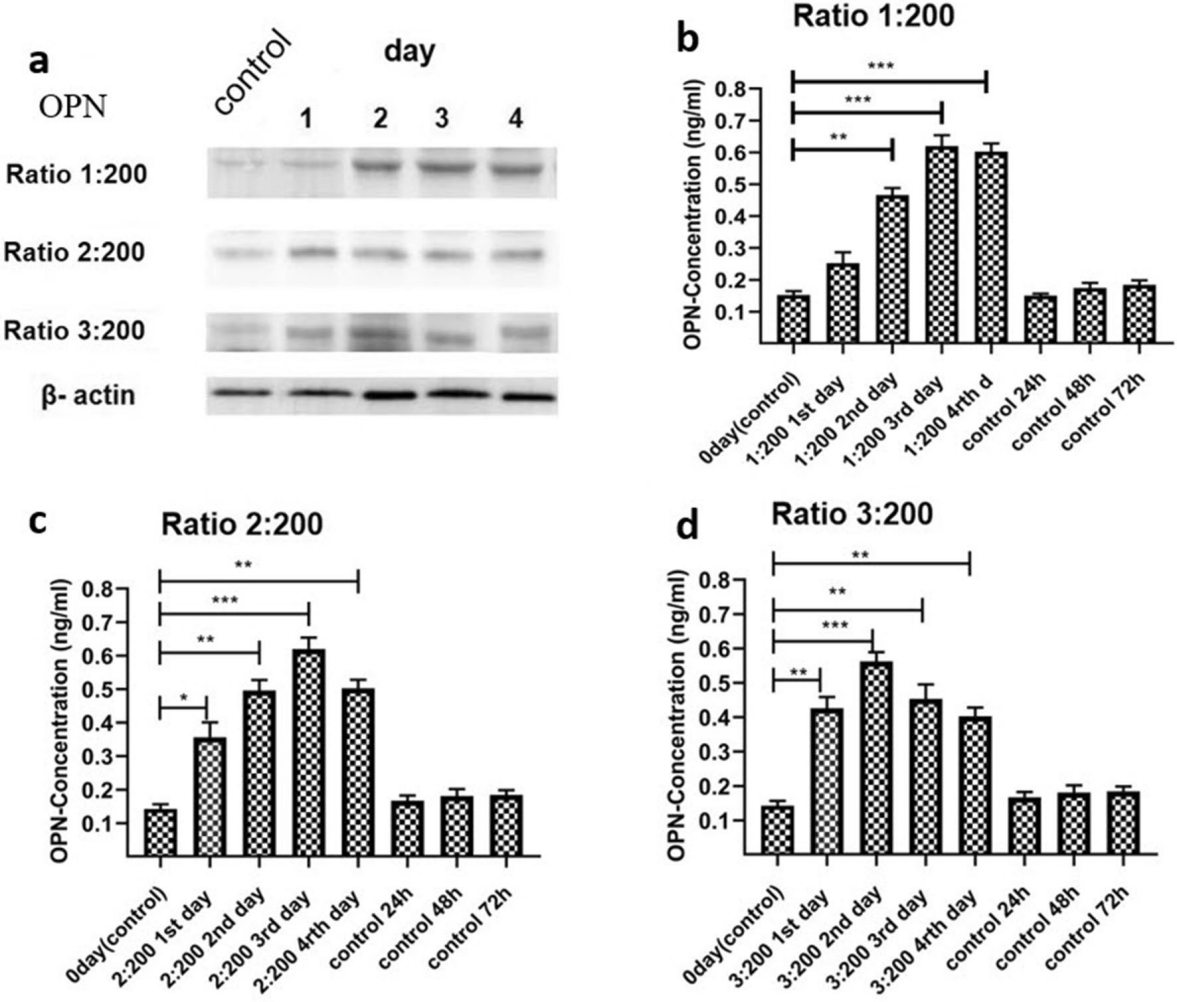
**a**–**d** Osteopontin (*OPN*) expression profile measured by WB and ELISA. **a** Expression of OPN assessed by WB. **b** OPN expression was significantly higher on days 2, 3 and 4, but not on day 1 day for the PSC:HSC-LX2 ratio of 1:200. **c** OPN expression was significantly higher on days 1, 2 and 3 at a PSC:HSC-LX2 ratio of 2:200. **d** At a PSC:HSC-LX2 ratio of 3:200, OPN expression had increased by days 1 and 2, but decreased by days 3 and 4. For other abbreviations, see Fig. 1

In addition to Col-I and α-SMA, OPN is also a major ECM protein produced during fibrosis. HSCs were incubated under coculture to identify the role of PSCs in OPN expression, which was measured by WB (Fig. 6a). OPN expression increased in the coculture of HSC-LX2 with PSCs during incubation for 4 days (Figs. 6b–d). The relationship at a PSC:HSC-LX2 ratio of 1:200 was significant on days 2 (*q* = 14.16, *p* < 0.01), 3 and 4 (*q* = 21.78, *p* < 0.001) (Fig. 6b, ANOVA: F_(4, 10)_ = 116.4, *p* < 0.0001); for a ratio of 2:200, the results were significant on days 1 (*q* = 8.11, *p* < 0.05), 3 (*q* = 13.83, *p* < 0.001), 2 (*q* = 13.43, *p* < 0.01) and 4 (*q* = 12.91, *p* < 0.01) (Fig. 6c, ANOVA: F_(4, 10)_ = 68.95, *p* < 0.0001). At a PSC:HSC-LX2 ratio of 3:200, the relationship was significant on days 1 (*q* = 12.29, *p* < 0.01), 2 (*q* = 18.20, *p* < 0.001) and 3 (q=13.47, *p*<0.001) and 4 (*q* = 11.48, *p* < 0.001) (Fig. 6d, ANOVA: F_(4, 10)_ = 90.42, *p* < 0.0001); however, OPN expression had increased by days 1 and 2, but decreased by days 3 and 4.

## Discussion

A cell culture model was designed using *E. multilocularis* PSC and HSC coculture in vitro to examine the mechanism of liver fibrosis in AE. We evaluated the potential interactions between HSC-LX2 and *E. multilocularis* PSCs by coculturing them in DMEM. Under coculture, PSCs of *E. multilocularis* activated HSC-LX2. Li et al. [23] showed that PSCs had rapid motility and were evaginated in the first days of a hepatocyte culture system. In the present study, HSCs exposed to PSCs did not contain lipid droplets, whereas the HSC controls did. The number of lipid-containing HSCs decreased significantly during the transformative phases of liver fibrosis, which indicated that they underwent fibrogenic transformation [24]. All of the features resulting from the exposure of the HSCs to the PSCs, including the division or malformation and irregularity of the nucleus, and the microvilli on the cell membrane increasing and the mitochondria swelling are indicative of apoptosis.

HSCs contain one or one more oval-shaped nucleoli. Morphological changes were observed in the nucleoli of cultured HSC in the form of multiple, thin elongated processes which extended from the cell body [25]. The activation of human HSCs results in the expression of α-SMA, which is a specific marker of this process [26]. Our study revealed that the levels of Col-I, α-SMA and OPN increased in HSCs when they were exposed to PSCs, and also increased in the supernatant. HSCs exposed to PSCs for 72 h, particularly at a high PSC:HSC-LX2 ratio, produced Col-I. This was also found in a study on the effect of *Echinococcus* spp. PSCs on the process of fibrosis [27]. Additionally, PSCs inhibited the expansion of HSCs by directly targeting TGF-βRI/II [28]. The fluid of the cysts that develop during cystic echinococcosis can inhibit the proliferation of HSCs and increase the levels of the main markers of HSCs, including Col-I and α-SMA [27]. Our study showed that PSCs stimulated HSC to secrete Col-I, α-SMA, and OPN. HSCs are typically dormant but develop into fibroblasts which accumulate in the ECM when the cells are activated during liver injury [8]. Thus, activation of HSCs is an important process in fibrosis. Collagen synthesis and α-SMA levels increased after different incubation periods of HSCs [8, 16]. A recent study reported that OPN can directly induce the activation and proliferation of HSCs, and that its expression may be either TGF-β dependent or non-dependent [29]. Our experiments confirmed that OPN expression increased in HSCs exposed to PSCs. The results of the present study and other studies suggest that HSCs can secrete OPN and that OPN secreted by other cells also activates HSCs.

Many studies have demonstrated the role of HSCs in the synthesis of ECM components, fibrosis and cirrhosis in bovine livers infected with helminths, e.g. *Fasciola hepatica* and *Dicrocoelium dendriticum*. HSCs play an important role in the development of fibrosis and other stages of cirrhosis [24, 30]. TGF-β plays a role in the differentiation of HSCs; it is the main factor that activates these cells, which leads to collagen deposition [31]. Fibrosis is a common, progressive pathological process that occurs after extensive liver injury. ECM deposits are characteristic features of liver fibrosis observed after HSC activation. During liver fibrosis, HSCs differentiate and become the main producers of components of the ECM [29, 32].

In their study of an in vitro model of liver fibrosis, Ren et al. found that *E. multilocularis* cyst fluid has a significant impact on messenger RNA levels of ERK1/2, JNK1/2 and p38 in rat HSCs, and also observed morphological changes in the HSCs, and karyopyknosis [33]. Gao et al. found that *E. multilocularis* infections could promote the proliferation of HSCs and cause an increase in Col-I and α-SMA levels in mice sera, and induce the deposition of collagen fibres in mice livers [34]. Wang et al. successfully cultured *E. multilocularis* PSCs from jirds to the larval stage, which produced extracellular vesicles in modified RPMI 1640 medium in vitro; the results of that work provided helpful insights for future research on the relationship between *E. multilocularis* PSCs and HSCs in vitro [35].

The findings discussed here show that the infective stage of* E. multilocularis* is a critical factor in the development of liver fibrosis in AE. The results of our study provide an experimental basis for further elucidation of the pathogenesis of liver fibrosis in AE.

## Conclusions

We explored the interaction between HSCs and *E. multilocularis* PSCs, and showed that the latter induced the activation of the HSCs, which then produced proteins that play a role in hepatic fibrosis (i.e. Col-I, α-SMA, and OPN). These findings may contribute to a better understanding of the pathogenesis of hepatic fibrosis in AE. Additional studies are needed that focus on the role of the PSCs of *E. multilocularis* in this complicated process to further understand the interaction between this parasite and its host.

## Data Availability

The datasets used in this study are available from the corresponding author on reasonable request.

## Abbreviations

AE: Alveolar echinococcosis
α-SMA: Alpha-smooth muscle actin
Col-I: Collagen-I
DMEM: Dulbecco’s modified Eagle’s medium
ECM: Extracellular matrix
ELISA: Enzyme-linked immunosorbent assay
HSCs: Hepatic stellate cells
OPN: Osteopontin
PSC: Protoscoleces
PVDF: Polyvinylidene difluoride
RT: Room temperature
SDS-PAGE: Sodium dodecyl sulfate polyacrylamide gel electrophoresis
TEM: Transmission electron microscopy
WB: Western blotting
